# Regional impacts of warming on biodiversity and biomass in high latitude stream ecosystems across the Northern Hemisphere

**DOI:** 10.1038/s42003-024-05936-w

**Published:** 2024-03-13

**Authors:** Michelle C. Jackson, Nikolai Friberg, Luis Moliner Cachazo, David R. Clark, Petra Thea Mutinova, Eoin J. O’Gorman, Rebecca L. Kordas, Bruno Gallo, Doris E. Pichler, Yulia Bespalaya, Olga V. Aksenova, Alexander Milner, Stephen J. Brooks, Nicholas Dunn, K.W.K. Lee, Jón S. Ólafsson, Gísli M. Gíslason, Lucia Millan, Thomas Bell, Alex J. Dumbrell, Guy Woodward

**Affiliations:** 1 Department of Biology, University of Oxford, Oxford, OX1 3SZ UK; 2 Georgina Mace Centre for the Living Planet, Department of Life Sciences, Imperial College London, Silwood Park Campus, Ascot, SL5 7PY UK; 3 Norwegian Institute for Nature Research (NINA) Sognsveien 68, Oslo, 0855 Norway; 4 Freshwater Biological Section, University of Copenhagen, Copenhagen, Denmark; 5 Water@Leeds, University of Leeds, School of Geography, Leeds, UK; 6 Department of Geography, King’s College London, The Strand, London, WC2R 2LS UK; 7 School of Life Science, University of Essex, Colchester, CO4 3SQ UK; 8 Institute for Analytics and Data Science, University of Essex, Colchester, CO4 3SQ UK; 9 The Norwegian Institute for Water Research (NIVA), Økernveien 94, Oslo, 0579 Norway; 10 N. Laverov Federal Centre for Integrated Arctic Research, Ural Branch, Russian Academy of Sciences, Arkhangelsk, Russia; 11 School of Geography, Earth and Environmental Sciences, University of Birmingham, Birmingham, B15 2TT UK; 12 Department of Life Sciences, Natural History Museum, Cromwell Road, London, SW7 5BD UK; 13 Institute of Marine and Freshwater Research, Hafnafjordur, 220, Hafnarfjörður, Iceland; 14 Institute of Life and Environmental Sciences, University of Iceland, Reykjavík, 102 Iceland; 15 Present Address: Kadoorie Farm and Botanic Garden, Lam Kam Road, Tai Po, Tsuen, Hong Kong

**Keywords:** Freshwater ecology, Biodiversity

## Abstract

Warming can have profound impacts on ecological communities. However, explorations of how differences in biogeography and productivity might reshape the effect of warming have been limited to theoretical or proxy-based approaches: for instance, studies of latitudinal temperature gradients are often conflated with other drivers (e.g., species richness). Here, we overcome these limitations by using local geothermal temperature gradients across multiple high-latitude stream ecosystems. Each suite of streams (6-11 warmed by 1-15°C above ambient) is set within one of five regions (37 streams total); because the heating comes from the bedrock and is not confounded by changes in chemistry, we can isolate the effect of temperature. We found a negative overall relationship between diatom and invertebrate species richness and temperature, but the strength of the relationship varied regionally, declining more strongly in regions with low terrestrial productivity. Total invertebrate biomass increased with temperature in all regions. The latter pattern combined with the former suggests that the increased biomass of tolerant species might compensate for the loss of sensitive species. Our results show that the impact of warming can be dependent on regional conditions, demonstrating that local variation should be included in future climate projections rather than simply assuming universal relationships.

## Abstract (web summary)

The responses of freshwater stream biodiversity and biomass to temperature in Arctic regions varies with biogeography.

## Introduction

Earth is warming rapidly, especially at higher latitudes in the Northern Hemisphere, and in combination with other anthropogenic activities (e.g. land-use change), this threatens biodiversity and ecosystem functioning across spatial scales^1–13^. However, responses to human activity among regions and biomes (e.g. Arctic tundra vs boreal forest), and ecosystem types (e.g. aquatic vs terrestrial)^1,5^ are often inconsistent, with a particular paucity of data on freshwaters, especially at high latitudes (but see ref. ^14,15^).

Previous studies have often used latitude as a proxy for temperature change, to infer the impacts of warming, but this approach is compromised by confounding variables (e.g. land-use, primary productivity). Since global species richness for most taxa also peaks near the equator and declines towards the poles^9,10^, and rising temperatures can increase species loss^16^, extrapolating the impact of temperature on ecosystems using latitudinal gradients is problematic for predicting future change^15^. Here, we quantified the effect of temperature within and among multiple regions at the biome scale for high-latitude freshwater ecosystems in the Northern Hemisphere to circumvent this longstanding research gap. Our approach combines the strengths of field observations with natural experiments by taking advantage of local geothermal warming (within regions) and large-scale (between regions) temperature gradients to determine whether thermal responses are consistent across different regions^17^. Within regions, the streams share the same river basin, with many only a few metres apart, but still varying in temperature of 1 to 15 ^o^C above ambient. Importantly, unlike many other heavily studied geothermal areas, such as Yellowstone National Park, our focal systems are warmed indirectly, from below the bedrock, so they do not suffer from the confounding effects of also being acid, enabling us to isolate the effects of temperature (see Supplementary Note 1).

**Fig. 1:**
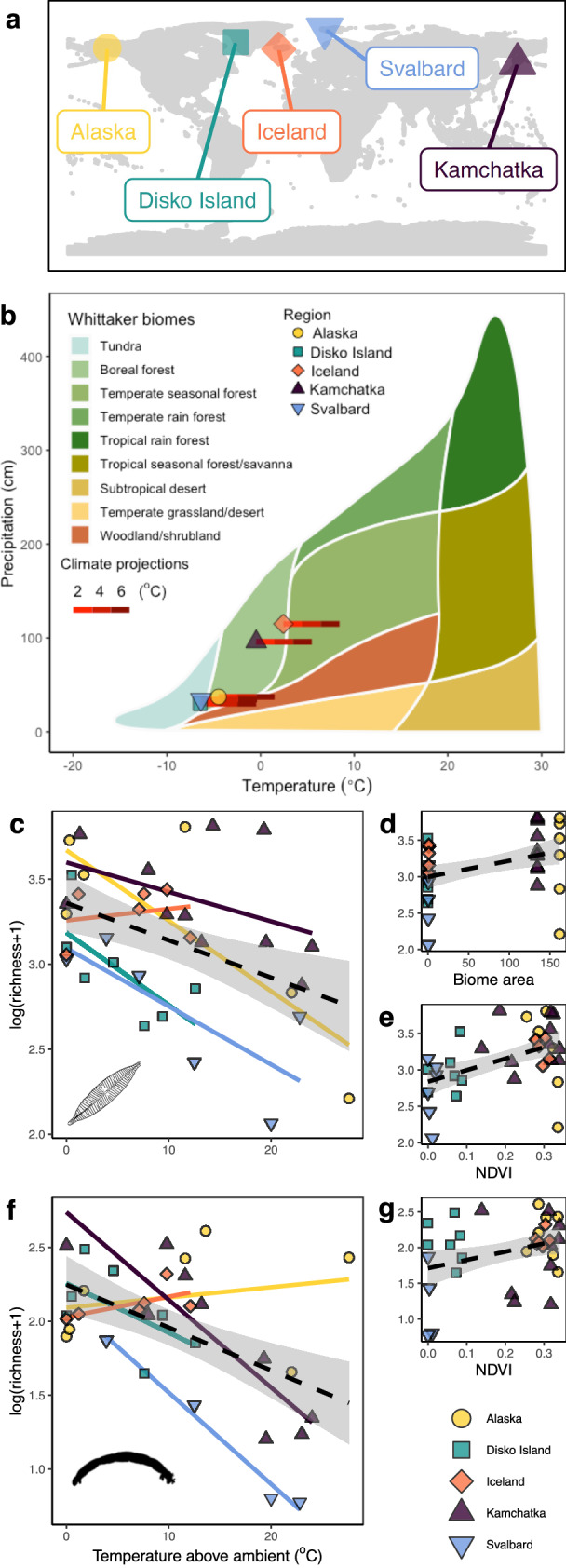
Study region map and Whittaker biome plot, and the effects of stream temperature and biogeography variables on species richness. **a** Location of the five regions and **b** the biome area each region occupies, including how this could be reshaped with climate projections. Temperature and precipitation are given as an annual average and total, respectively. This figure was made using the *plotbiomes* package in R (https://valentinitnelav.github.io/plotbiomes/). **c** General linear model’s showed that diatom richness was predicted by a combination of temperature, **d** biome area (in millions of km^2^), **e** and NDVI. **f** For invertebrates, richness was predicted by temperature, and **g** NDVI. Symbols in **c**–**g** represent a single stream, coloured lines are the linear model for each region, and the dashed black line represents the overall linear trend with 90% confidence intervals.

Freshwaters in the colder boreal to Arctic biomes may be particularly vulnerable to warming and loss of species richness^2,18^, and our five regions—Iceland, Svalbard (Norway), Kamchatka (Russia), Alaska (USA), and Disko Island (Greenland; Fig. 1a)—span these Northern Hemisphere biomes and their different biogeographical (Fig. 1b) and phylogenetic histories. These regions differ in (i) biome area (the area of the ecological biome before encountering a sea barrier), (ii) isolation (distance to the next nearest landmass) and (iii) terrestrial plant biomass (Normalised Difference Vegetation Index [NDVI] for the year prior to sampling as a proxy; see Methods), allowing us to unravel context-dependencies in temperature effects on community and ecosystem properties.

One such context-dependency is remoteness, with Island Biogeography Theory^19,20^ predicting that species richness should decrease more rapidly in smaller and more isolated regions (e.g. islands versus continents) due to the smaller regional species pool, with fewer habitats and limited dispersal opportunities across the metacommunity^21–23^. This is partly due to lower turnover rates (i.e. the variation in community membership among neighbouring sampling locations) in islands when compared to larger and less isolated regions (i.e. continents) because of the greater regional species pool and, therefore, scope for the replacement of species that are better adapted to the warmer conditions^24,25^.

Another body of ecological theory, based on metabolic constraints, suggests that the impact of warming on diversity will vary with primary productivity^26^, so both community and ecosystem responses could vary regionally if there is a variation in regional productivity. For instance, meeting the higher metabolic demands imposed by a warmer environment becomes harder when resources are limited, so (all else being equal) this should limit the number of thermal niches and hence the number of species an ecosystem can support^27^. Alternatively, smaller body sizes might allow organisms to reduce their metabolic demands and persist in warmer environments^27,28^. Therefore, warming should generally favour smaller species, although recent research suggests its effects in stream ecosystems can be mitigated if resources are sufficiently plentiful^28,29^, such that more productive systems may be better buffered against climate change than previously thought.

Most of the research on temperature gradients has focused on species richness, with few studies of other responses which are linked more directly to ecosystem functioning (e.g. community biomass) at large spatial scales, and fewer still have assessed both biomass and richness from the same ecosystems (but see ref. ^30^). Individual total metabolic rates increase with warming and, therefore, if resource supply remains constant, we expect total community biomass to decline (since each individual will need to use a larger proportion of the total energy available). However, we currently do not know if warming has the same impact on both community composition and ecosystem functioning or if community and ecosystem responses to warming are shaped by their regional context.

At large scales, the major global biomes within which each local ecosystem is embedded are defined by their terrestrial vegetation, which in turn is driven by temperature and precipitation (Fig. 1b). We selected sites across five regions that span a gradient of the first of these fundamental axes—temperature (see Methods)—representing contemporary Arctic and Boreal biomes (classified using standard Whittaker biomes), with the second biome axis associated with water limitation being effectively factored out at the local scale by using freshwaters as our study systems. Therefore, each region has a characteristic thermal profile with different terrestrial vegetation, providing the template onto which we consider additional local temperature gradients in the focal streams^31–33^. To provide some context to the scale of change that this equates to, with future regional warming of just 2–6 ^o^C, all of the five regions would effectively move into a new biome classification (e.g. from tundra to boreal, from boreal to temperate seasonal forest etc). These biome-level shifts in temperature regimes map onto future global warming scenarios, and fall well within the common Intergovernmental Panel on Climate Change projections for 2100^11^ (Fig. 1b): for instance, the temperature gradient across the streams (imposed by geothermal activity) in our Svalbard sites would equate to a shift from Arctic tundra to woodland conditions, which is widely forecast to occur by the end of this century^11^. Our temperature gradient also goes beyond IPCC predictions to determine potential non-linearities and the rate of change.

This nested study design allowed us to test the following three principal hypotheses;

(1a) Species richness (alpha diversity) will decline with increasing water temperature, and (1b) this trend will be strongest in regions with low productivity (i.e. Arctic tundra compared to more productive boreal forest). Since annual secondary production is logistically challenging to measure directly, especially in these remote environments, we used regional terrestrial vegetation as a proxy of regional productivity, because freshwaters mirror the productivity of the landscape within which they are embedded^34,35^. (2a) Species turnover within regions will increase with pairwise stream temperature differences, and (2b) turnover will be higher in small, isolated regions.

(3a) Community biomass will decrease with increasing stream temperature, and (3b) this trend will be strongest in regions with low productivity. We used standing community biomass of consumers within each stream as our focal ecosystem-level response, since this drives many key ecosystem processes and is also often strongly correlated with secondary production^36,37^.

## Results and discussion

**Fig. 2:**
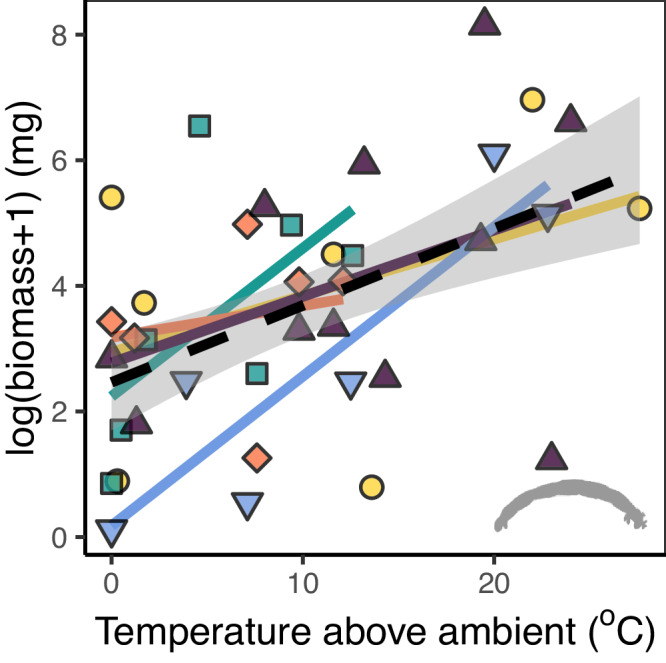
Effects of stream temperature on invertebrate biomass. Our general linear model’s showed that biomass consistently increased with temperature in all five regions regardless of biogeography. Symbols represent a single stream, coloured lines are the linear model for each region, and the dashed black line represents the overall linear trend with 90% confidence intervals.

Overall, temperature had effects on both species richness and biomass, but the strength, and in some cases the direction, varied among regions. In support of hypothesis 1a, species richness significantly declined with water temperature when considering all sampled streams across all regions (Fig. 1, Table 1, and Supplementary Table 2). At smaller spatial scales, context-dependencies become more apparent: richness generally declined with warming but with some exceptions in regions with low terrestrial productivity (supporting hypothesis 1b). In contrast to the negative relationship with richness, invertebrate biomass (i.e. secondary production) increased with water temperature in all five regions (Fig. 2 and Table 1), contradicting the theory that richness supports functioning^37–39^. This also contradicted our hypothesis 3, based on standard metabolic theory, that temperature would cause a decline in biomass. Since biomass is more directly linked to many ecosystem processes (such as decomposition and productivity) than is true for biodiversity metrics like richness and turnover^38,39^, this suggests that species loss with warming might not necessarily lead to loss of functioning. Here, the increased biomass of tolerant species might compensate (at least partially) for the loss of biomass in more sensitive species.

**Table 1:** Species richness and total biomass

	Response	Model	df	*F*	*P*
	Diatom richness	Temperature	1	13.42	0.001
GLM		Region	4	8.01	<0.001
		Temperature*Region	4	1.12	0.368
	Invertebrate richness	Temperature	1	26.81	<0.001
		Region	4	6.97	0.001
		Temperature*Region	4	6.53	0.001
	Invertebrate biomass	Temperature	1	10.19	0.004
		Region	4	0.56	0.696
		Temperature*Region	4	0.61	0.658
Optimum	Diatom richness	Temperature*NDVI*Area	1,28	1.55	0.223
	Invertebrate richness	Temperature*NDVI	1,29	11.06	0.002

General linear models (GLM) revealed a significant effect of water temperature on all response variables, but region only had an effect on species richness. We also found a significant interactive effect of temperature and region on invertebrate richness, but not diatom richness or invertebrate community biomass. Where region had a significant effect, we explored this further by testing for interactive effects of temperature and biogeography variables (only optimum models are shown; see model selection in Supplementary Table 2).

**Fig. 3:**
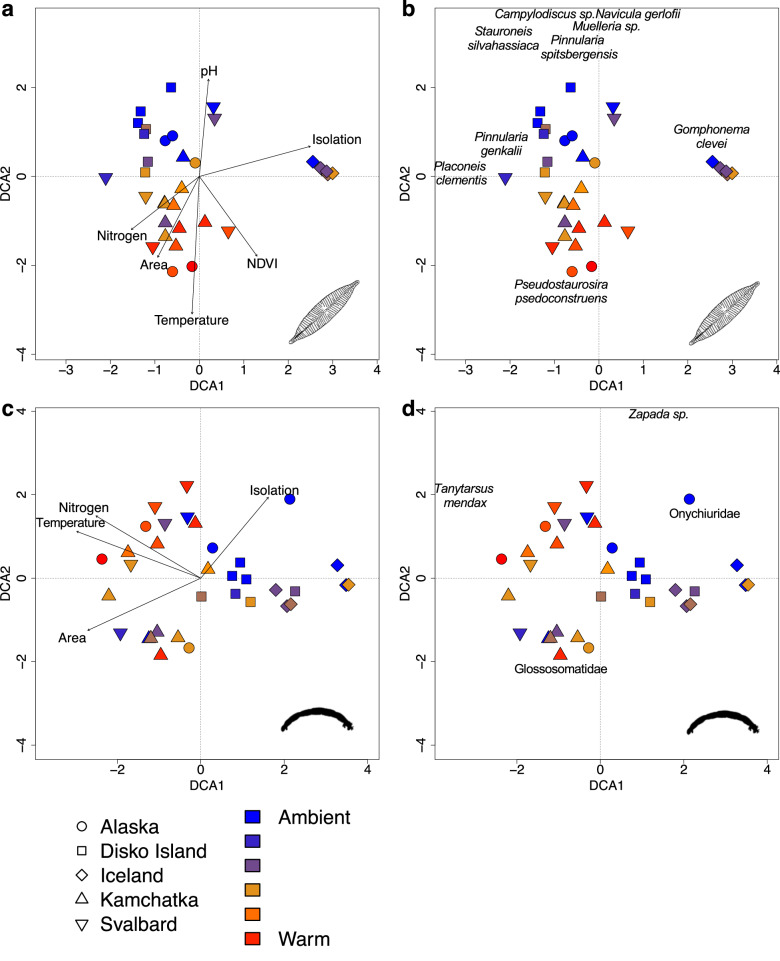
Effects of study region and stream temperature on community composition (relative abundance). Detrended correspondence analysis plots are shown for all diatom (**a**, **b**) and invertebrate (**c**, **d**) species, where each point represents the community in a single stream. Symbols represent the five regions and colours scale from ambient cold (blue) to warm (red) streams. Plots either show passively overlaid environmental variables (**a**, **c**), or important species which vary between the communities (**b**, **d**). Full results are in Supplementary Table 5.

Species turnover increased with water temperature (supporting hypothesis 2a), but this was only significant in Kamchatka (invertebrates and diatoms) and Iceland (invertebrates). Indeed, the slopes describing turnover-temperature relationships varied regionally (Supplementary Fig. 4 and Supplementary Table 3), but in contrast to hypothesis 2b, the variation in slopes was independent of the focal biogeographical variables (Supplementary Table 4). However, Detrended Correspondence Analysis (DCA) revealed some separation between the communities in the Arctic tundra (Disko Island, Svalbard) and boreal forest (Iceland, Alaska, Kamchatka) regions. As predicted, there was also an effect of isolation—Iceland, the most isolated region, had the most distinct diatom and invertebrate communities in our ordinations (Fig. 3). This variation among regions was correlated with our focal geographical variables (isolation, biome area, NDVI; Fig. 3a, c) and, although water temperature had an effect in structuring communities, this was much stronger within each region than among regions (Fig. 3b, d and Supplementary Table 5). These results imply that future warming may have profound effects on community composition at local scales. Despite these strong effects of warming, other stream variables were also important, but their influences were weaker than temperature. Our DCA’s showed that dissolved inorganic nitrogen was a significant variable in structuring both diatom and invertebrate communities, while pH was only significant for diatoms (Fig. 3 and Supplementary Table 5). However, the amount of variation explained by temperature was always higher (Supplementary Table 5), indicating that this is the ‘master variable’ in these streams.

Temperature and region both independently affected species richness, and they also had interactive effects on invertebrate richness but not diatom richness, (Table 1 and Fig. 1). In all five regions, stream temperature alone explained 23% of the variation in invertebrate richness and 15% of diatom richness. Adding specific biogeographical variables for each stream (instead of just using the region as a single categorical descriptor) significantly improved our models’ predictive power (Supplementary Table 2), with diatom species richness best explained by a three-way interaction among water temperature, terrestrial productivity, and biome area (54% of variation). Diatom richness generally decreased with temperature (except in Iceland; Table 1, Supplementary Table 2, and Fig. 1c) but increased with biome area and productivity (hypothesis 1a; Fig. 1d, e). Although ref. ^40^ did not isolate the effect of temperature as we did here, they also found that global freshwater diatom richness was partially explained by climate, area, and productivity, with the specific trends varying between continental and island sites^40^. Invertebrate richness was best explained by both temperature and terrestrial productivity (52% of variation; Fig. 1f, g). Specifically, temperature had a negative effect except in the regions with the highest terrestrial productivity (Alaska, Iceland; Table 1, Supplementary Table 2, and Fig. 1f). Again, although other studies have found an important influence of climate variables on invertebrate species composition in high-latitude regions^41,42^, these were not able to directly attribute variation to temperature alone. By using suites of streams varying in temperature within regions, we can conclude that water temperature is the main driver of change. In these high-latitude locations, the regional species pool will almost certainly have more cold- (as opposed to warm-) adapted diatoms and invertebrates, given that most of the surrounding landmass is colder than our warmed systems, which seems a likely explanation for why richness is generally lost with temperature.

In contrast to our third hypothesis, the water temperature had a significant positive and geographically consistent (Fig. 2 and Table 1) effect on invertebrate biomass (i.e. the effect of the region itself was not significant). Theory^30^ and evidence from controlled experiments^27^ predict that biomass should be temperature-invariant, or should decline if resources are limited. The different result observed here mirrors findings from our previous work in Iceland where we found that fish and invertebrate biomass actually increase with temperature^14,28^. This might be because invertebrates were at the lower end of their thermal limits in the colder streams, with the warmer temperatures bringing them closer to their thermal optimum^14^. Alternatively, if the nutrient supply rate increases with temperature (as we showed in a previous study in our Icelandic sites^26^), this can support faster ecosystem processes at the base of the food web and hence a larger than otherwise expected biomass of consumers. Since nitrogen concentration was not depleted with temperature in our streams (Supplementary Fig. 3e), our results suggest similar mechanisms might indeed be at play more widely across the Northern Hemisphere. In other words, if resources remain plentiful across the temperature gradient, consumers may be able to keep up with their rising metabolic demands and gain more total biomass with warming. Given the logistic and financial constraints of conducting such a large-scale study that spans the Northern Hemisphere, we were constrained to a single field campaign that captured the peak growing season, due to the remoteness of most sites. Potentially, future work could explore temporal (seasonal and interannual) fluctuations in both richness and biomass, if these constraints can be overcome.

Overall, our results show that temperature increased turnover within regions while decreasing species richness. Declines in species richness were partially offset by the effects of terrestrial productivity, supporting a recent global meta-analysis suggesting local extinction was more common at high latitudes^43,44^ (where terrestrial production tends to be low). The declines in invertebrate species richness contrasted with increases in overall invertebrate biomass, suggesting that the two sides of the biodiversity-ecosystem functioning relationship are not necessarily both negatively affected by warming and that compensatory mechanisms might be operating, such that even if species are lost, the community biomass that drives many key functions and processes could actually rise. We argue that both species richness and biomass need to be considered to fully understand the effects of climate warming.

Understanding and predicting how communities and ecosystems will respond to future climate change remains one of the greatest challenges in ecology. Here, we have shown that species richness in streams generally declines with temperature, but that this is not universal. Therefore, regional granularity will be needed for making future projections, especially given the potential scope for compensatory biomass vs richness responses: coarse one-size-fits-all approaches are clearly inadequate for scaling up from local to global forecasts of impacts on both biodiversity and ecosystem properties.

## Methods

### Location

Our study was conducted in five high-latitude regions (Fig. 1a) with geothermal areas nested within them to provide natural thermal gradients: Iceland (mean summer stream temperature of sampled streams: 5.8–20.3 °C), Kamchatka (Russia; 7.0–31.0 °C), Alaska (USA; 5.5–33.1 °C), Disko Island (Greenland; 1.7–14.3 °C), and Svalbard (1.5–24.3 °C; Supplementary Table 1). Within each region, all streams share the same river basin (see Supplementary Note 1). We sampled 6–11 streams per region, with the coldest stream in each considered as the baseline water temperature ‘control’. The streams across all regions were very similar in their physical characteristics (width, depth, see Supplementary Fig. 1 and Supplementary Table 1) and occurred in pristine landscapes, with no pollutants from anthropogenic activities. All streams were groundwater‐fed and hydrologically stable^17^. There were no confounding effects of water chemistry across the temperature gradient^17^ except a weak correlation of temperature with pH in Svalbard (See Supplementary Note 1, Supplementary Table 1, and Supplementary Figs. 2, 3). The pH variation was lower than the range of values recorded annually in most single freshwater systems^43^ and, therefore, we are confident that the responses we describe are due to temperature and not pH.

The regions varied in terms of terrestrial productivity (Supplementary Table 1) and connectedness to continental sources of diversity (i.e. islands vs continents). We visited each region in August 2013 (except Iceland, which was visited in August 2012) and, at each stream, measured water temperature, pH, and conductivity using a water quality metre at a minimum of five points to calculate average stream conditions. We also took water samples for chemical analysis. These were filtered through a Whatman GF/C filter mounted on a syringe and collected in a 100 ml polyethylene bottle. 1% sulphuric acid (4 M H2SO4) was added to preserve the sample. Analysis was subsequently undertaken in the University of Aarhus water quality laboratory using Perkin Elmer 4100 apparatus and the Danish Standard 221 protocol for Total bioavailable N^45^. Due to the remote location of most of the regions, we were only able to take point measurements^17^.

### Community analysis

We sampled benthic invertebrates from each stream using a Surber sampler (0.0225 m^2^, 250 um mesh). Three technical replicate samples were collected from random locations in each stream and preserved in 70% ethanol^28,30^. All invertebrates were identified and counted using a Nikon SMZ800 at a magnification of X10-63. Diatoms were sampled by randomly selecting three stones which were then brushed using a toothbrush detaching algae while rinsing with a spray bottle using water^28^. Algae samples were preserved using a few drops of 5% Lugols solution. Slides were prepared by the standardised method^46^ of chemical digestion by hydrogen peroxide (H_2_O_2_, 30%) and mounting into the synthetic resin Naphrax (Brunel Microscopes Ltd. Wiltshire, UK). From each sample, 400 randomly encountered diatom valves were determined to the lower taxonomical level possible using a Leica DM2500 light microscope, at a magnification of 1000^46^. Identification was performed by several researchers (two for diatoms, four for invertebrates), and therefore, a process of data harmonisation was performed to ensure consistency (see Supplementary References for identification guides).

Next, we determined the total invertebrate biomass in each stream by first estimating the average invertebrate species body masses. Here, a single linear dimension was measured for 1–150 individuals [average 14] of each species in each stream, and individual biomass was estimated using published length-mass equations^28^. Note that in a small number of cases, species body mass was estimated from streams of similar temperature within the same region. Total biomass was then calculated by multiplying the average species body mass of each taxa in the community by the population abundance.

### Biogeographical variables

All analyses were conducted in R version 4.2.1 (R Core Team 2019). For each region, we calculated two biogeography variables: (i) biome area (the area to which the region’s biome extends before encountering a sea barrier), and (ii) isolation (shortest distance to nearest continental landmass) Isolation was considered 0 km for continental sites (i.e. they are 0 km away from the nearest continent). For each of the 37 streams, we also calculated surrounding terrestrial plant standing stock (using the average normalised vegetation index; NDVI). We downloaded NDVI data from the MOD13Q1 product at 250 m spatial resolution (16-day composited data) using the *MODISTools* R package version 1.1-4^47^. We used the annual average NDVI taken for the full year before the sampling month (August 2012 or 2013) to capture the annual terrestrial production in the landscape in which the streams are embedded. The area around each site that was considered was 250 m^2^ (with the sampling location at the centre).

### Statistics and reproducibility

Firstly, we calculated rarefied species richness (i.e. alpha diversity) based on the smallest sample size across pooled replicate samples (400 individuals for diatoms; 43 individuals for invertebrates after removing four streams with very low abundance (<35)) in the *vegan* package version 2.6-2^48^. We also calculated Sørensen dissimilarity between streams in each region, and partitioned this into two components of beta diversity using the *betapart* package version 1.5-6; nestedness and turnover^49,50^. Turnover contributed >99% in all pairwise comparisons, so we only use the turnover component of beta diversity.

Next, we used bootstrapped (1000 bootstraps) exponential decay models to characterise relationships between temperature (above ambient) and community turnover^47^, from which we calculated average slopes for each region. We tested for the effects of our biogeographic variables (NDVI was averaged for each region) on the temperature-dissimilarity slope for each region using standard linear regression. Variation in community structure was further explored using Detrended Correspondence Analysis (DCA) in the *vegan* package^48^. We used square root transformed relative abundance data to dampen the influence of outliers and passively overlaid the effect of environmental variables (temperature, pH, conductivity, nitrogen, biome area, isolation and NDVI).

To test for an effect of temperature (above ambient), region, and their interaction on alpha diversity and invertebrate biomass, we used general linear models after transforming data (log(x + 1)). The region had an effect on species richness (see Results) so to explore this further we compared 19 models with stream temperature (above ambient), stream dissolved inorganic nitrogen, average NDVI, isolation, and biome area as our predictor variables (independently, and in all possible pair and three-way interactive combinations (except for isolation * biome area due to collinearity, Supplementary Fig. 2). The optimal model was selected using Akaike Information Criterion corrected for small sample sizes (AIC_c_; Supplementary Table 2) and the significance of these models was also tested by comparing output with a ‘null’ model, where we removed all fixed effects but the intercept.

### Reporting summary

Further information on research design is available in the Nature Portfolio Reporting Summary linked to this article.

### Supplementary information

Supplementary Information

Reporting Summary

## Data Availability

All data and have been uploaded to FigShare: https://portal.sds.ox.ac.uk/projects/Little_Ring_of_Fire_WP1/195952
