# Supplementary material for: Regional impacts of warming on biodiversity and biomass in high latitude stream ecosystems across the Northern Hemisphere
Source: Commun Biol. 2024 Mar 13;7:316. doi: 10.1038/s42003-024-05936-w (PMC10937648; doi:10.1038/s42003-024-05936-w)
Supplement: Supplementary file 1 — Supplementary Information [file 42003_2024_5936_MOESM1_ESM.pdf]

Regional impacts of warming on biodiversity and biomass in high latitude stream ecosystems across the Northern Hemisphere

Jackson et al. **Supporting Information**

Supplementary Note 1: STUDY SYSTEM

Geothermal regions offer ideal testbeds for investigating how warming can alter ecosystems. Hengill, a geothermal region in Iceland (hereafter, Iceland), has been used as a model system for conducting such "natural experiments" over the past decade to make predictions about future climate change over multiple organisational levels, from individuals to ecosystems[1]. Here, freshwater invertebrate abundance increases with temperature, while richness increases about to about 10°C above ambient[2]. Diatoms decrease in abundance with temperature, but in general biomass stocks across all tropic levels and key ecosystem processes such as primary and secondary production, nutrient cycling and decomposition rates increase with this level of warming[3–5]. In the current paper we tested the generalities of these findings by expanding these model systems to include analogues across the Norther Hemisphere in four other high-latitude regions - Alaska (USA), Disko Island (Greenland), Kamchatka (Russia), and Svalbard - which vary in biogeography and biome type (Supplementary Figure 1, Supplementary Table 1). A paired paper (Clark et al., in prep) considers how microbial communities vary in a subset of these streams across the five regions (Supplementary Table 1). Clark et al. also have 5 streams from Iceland which were not sampled for our paper.

Supplementary Figure 1: *Site photos. Alaska* ***(a)****, Disko Island* ***(b)****, Iceland* ***(c)****, Kamchatka* ***(d)****, and Svalbard* ***(e)****. Photos by Nikolai Friberg and Eoin O'Gorman. Researcher faces were edited from d to maintain anonymity.*

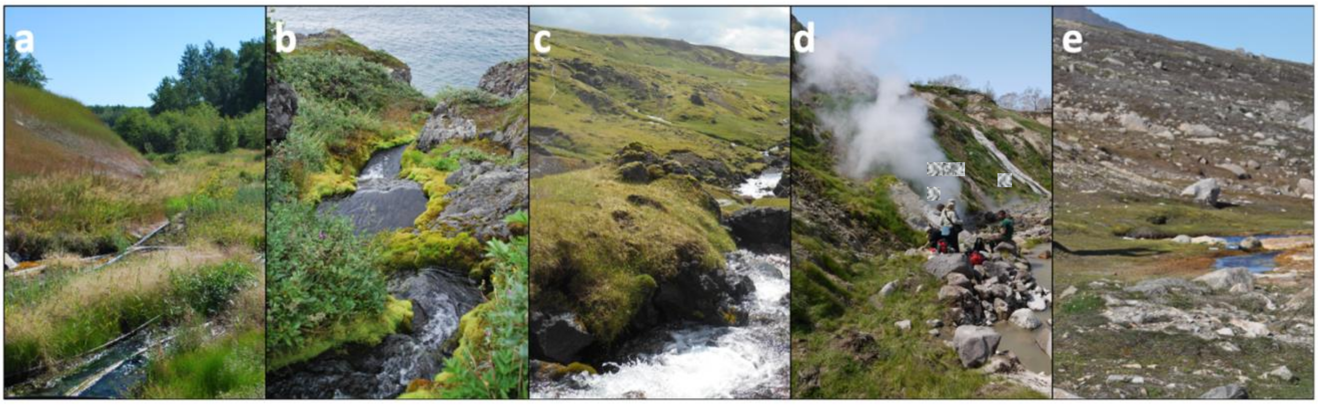

Supplementary Table 1: *Site details*

Code	Location	Stream temperature (ºC)	NDVI	pH	Conductivity (uS/cm)	Dissolved Inorganic Nitrogen (ug/ml)	Total Phosphorus (ug/ml)	DOC (ug/ml)	Width (m)	Depth (m)	Latitude	Longitude	Included in paired microbial paper?
A1	Alaska	33.1	0.34	7.86	552.00	1.30	0.036		1.75	0.4	65.0046	-150.6321	
A13	Alaska	5.5	0.32	6.68	179.90				2	0.9	65.0618	-150.6049	
A2	Alaska	27.5	0.34	7.34	459.00	0.66	0.084		0.9	0.1	65.0044	-150.6308	Yes
A6	Alaska	5.8	0.25	7.35	45.20	0.82	0.026		1	0.3	64.9952	-150.6544	Yes
A7	Alaska	17.1	0.31	7.56	487.00	0.34	0.107		0.5	0.15	65.0018	-150.6376	Yes
A8	Alaska	19.1	0.29	7.32	709.00				1	0.1	65.0065	-150.6191	Yes
A9	Alaska	7.2	0.29	7.97	292.00	1.33	0.035		1	0.2	65.0065	-150.6191	
G1	Disko Island	3.5	0.07	9.26	73.50	0.41	0.025		1.5	0.1	69.2647	-53.4397	
G12	Disko Island	11.1	0.00	9.39	107.10	0.46	0.018		1	0.2	69.4431	-53.8956	Yes
G14	Disko Island	1.7	0.06	9.48	70.90	0.37	0.020		0.35	0.3	69.2506	-53.5216	
G2	Disko Island	14.3	0.09	7.88	970.00	0.65	0.008		0.5	0.05	69.2594	-53.5686	Yes
G5	Disko Island	2.2	0.08	7.52	125.10	0.49	0.115		0.5	0.1	69.5747	-53.9897	Yes
G8	Disko Island	6.3	0.00	8.22	64.40	0.50	0.025		2.3	0.1	69.5539	-53.5244	Yes
G9	Disko Island	9.3	0.07	8.22	1755.0	0.36	0.003		1.5	0.1	69.5561	-53.6058	
K1	Kamchatka	31	0.22	8.30	650.00	0.32	1.060	3.88	0.35	0.03	54.4383	160.1411	
K10	Kamchatka	20.2	0.34	7.40	464.00	0.72	0.260	1.80	0.75	0.12	52.9	158.2	Yes
K11	Kamchatka	7	0.31			1.27	0.210	1.72	0.8	0.13	52.8	158.1656	Yes
K14	Kamchatka	8.3	0.32	7.30	90.00	1.28	0.162	1.40	0.75	0.008	52.79	158.1639	
K15	Kamchatka	15	0.32	7.00	256.00	1.12	0.179	1.68			52.84	158.1653	Yes
K18	Kamchatka	16.8	0.14	7.00	89.00	1.47	0.164	2.03	0.3	0.04	52.7	158.1	
K19	Kamchatka	18.6	0.34	7.10	110.00	1.48	0.148	1.23	0.6	0.06	52.85	158.15	
K20	Kamchatka	26.3	0.32	7.50	348.00	0.97	0.159	1.80	0.5	0.075	52.8236	158.14	Yes
K4	Kamchatka	21.3	0.19	6.80	42.00	1.67	0.129	2.19	0.75	0.035	54.4	160.1414	
K5	Kamchatka	30	0.22	5.90	982.00	3.00	0.420	0.98	1.25	0.1	54.39	160.1436	
K6	Kamchatka	26.5	0.32	6.90	256.00	0.21	0.520	2.74	20	1.25	52.1075	157.655	
S1	Svalbard	21.5	0.01	6.90	3810.0	1.02	0.015		0.5	0.03	79.3972	13.4408	Yes
S2	Svalbard	8.6	0.02	8.20	184.00	0.65	0.008		0.3	0.05	79.3931	13.4	Yes
S4	Svalbard	14	0.00	7.40	430.00	0.63	0.012		0.25	0.03	79.38	13.4414	Yes
S6	Svalbard	1.5	0.02	8.60	134.00	0.58	0.005		0.4	0.05	79.4011	13.4414	Yes
S7	Svalbard	24.3	0.00	7.20	1604.0	0.74	0.006		0.75	0.08	79.46	13.2902778	Yes
S8	Svalbard	5.4	0.00	7.30	648.00	0.80	0.006		0.3	0.07	79.46	13.2847	Yes
I11_12	Iceland	8.1	0.28	8.00	624.00	0.05			0.46	0.06	64.0574	-21.3115	
I13_12	Iceland	6.9	0.30	7.60	201.00	0.05			1.47	0.08	64.0467	-21.2865	
I14_12	Iceland	14.5	0.28	8.10	254.00	0.05			1.27	0.12	64.0593	-21.3187	
I3_12	Iceland	16.7	0.30	7.90	275.00	0.04					64.0555	-21.3038	Yes
I6_12	Iceland	19	0.31	8.10	283.00	0.00			0.8	0.11	64.0558	-21.3066	
I9_12	Iceland	14	0.29	8.10	262.00	0.04			0.63	0.06	64.0569	-21.3081	

In each location we sampled 6-11 streams in August 2012 (Iceland) or 2013 (all other sites). The maximum distance between streams in each location was 850m in Iceland, 2000m in Alaska, 3500m in Greenland, 5000m in Svalbard, and 300km in Russia (the only site with streams in 2 catchments). A Pearson's correlation revealed an overall significant relationship between temperature and pH (correlation -0.43; Supplementary Figure 2), but this was largely driven by Svalbard as an outlier (Supplementary Figure 3) and all other sites had similar pH across the temperature gradient. However, we did not use pH in any of our models (Supplementary Figure 2). We also checked for collinearity between all of our predictor variables, including our biogeographical variables (Supplementary Figure 2). There was a strong correlation between biome area and isolation (-0.74, Supplementary Figure 2), so we did not use these two variables together in any models.

Supplementary Figure 2: *Correlation matrix for our predictor variables. region_area = area occupied by biome; NDVI = normalized vegetation index (terrestrial productivity); Mainland = distance from mainland as a measure of isolation. Only significant correlations (p<0.05) are shown, with colour indicating if the relationship is positive or negative.*

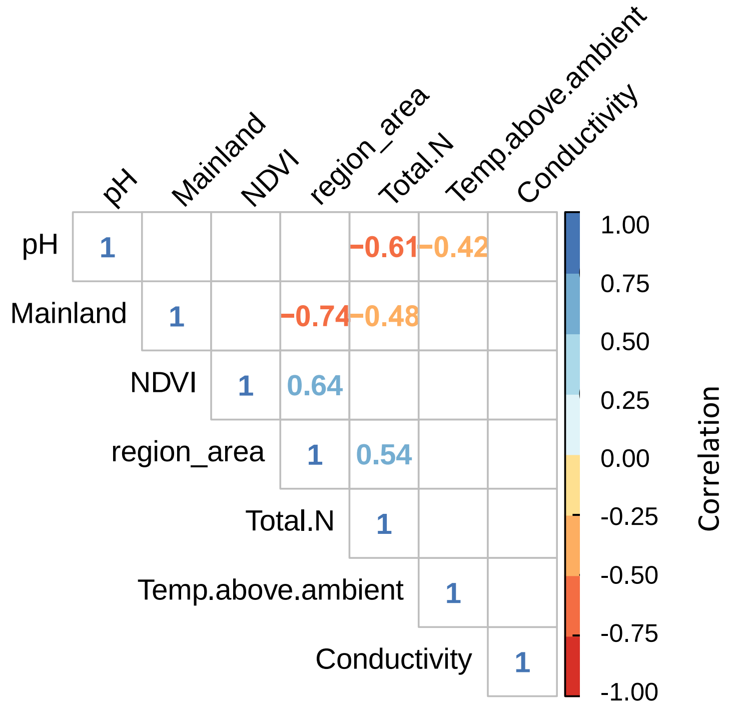

Supplementary Figure 3: *Relationship between stream temperature (above ambient) and other characteristics:* ***(a)*** *pH,* ***(b)*** *conductivity,* ***(c)*** *dissolved inorganic nitrogen (DIN),* ***(d)*** *NDVI,* ***(e)*** *biome area, and* ***(f)*** *isolation.*

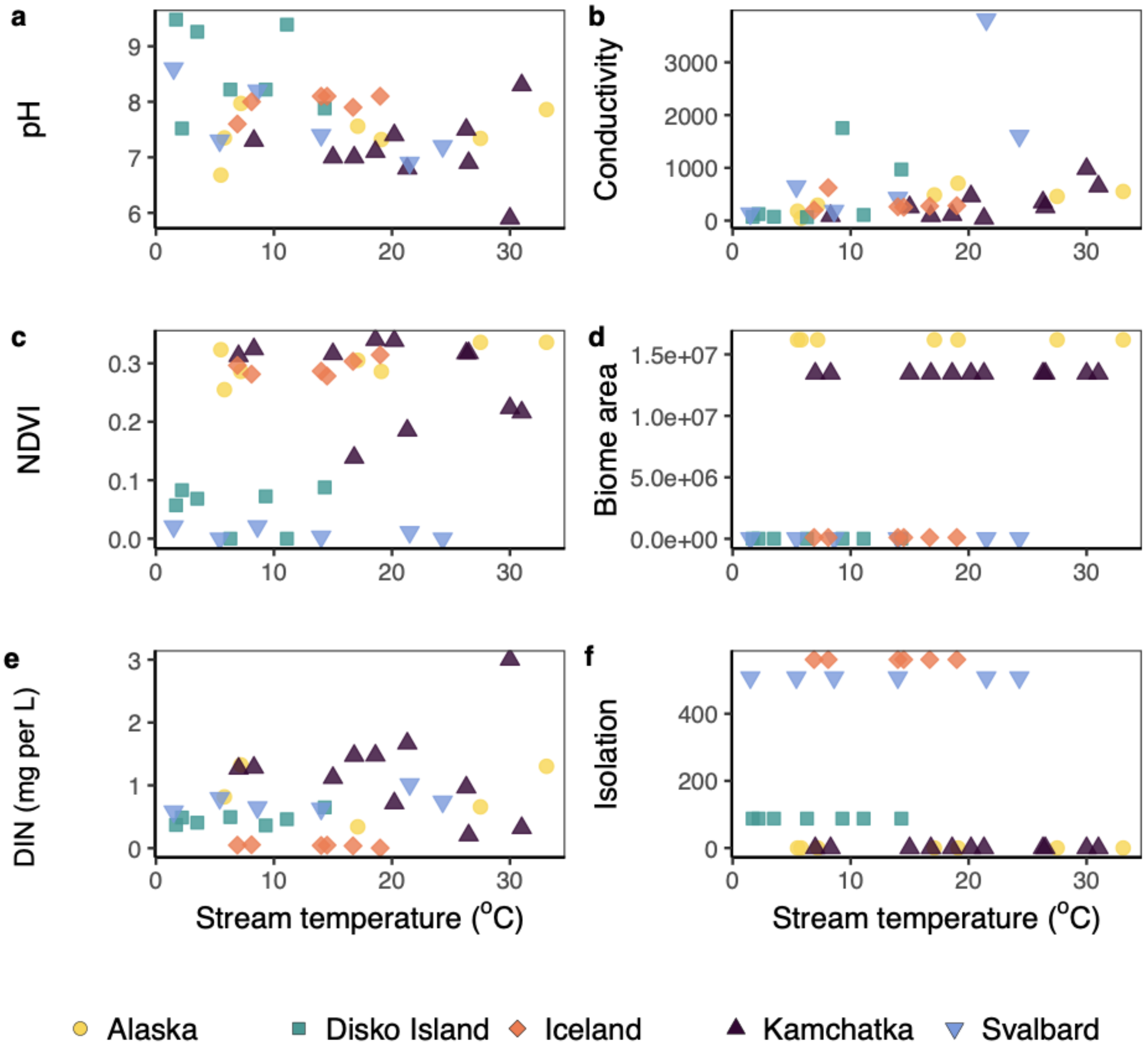

FURTHER RESULTS

Supplementary Table 2: *Best fit model selection for the effect of temperature and biogeography on stream species richness (alpha diversity). The best model is highlighted in blue.*

Alpha diversity	Predictors	AICc	RSE	R^2(adj)
Diatoms	Intercept only	44.730	0.430	NA
	Temperature above ambient	40.115	0.396	0.153
	Nitrogen	46.883	0.442	0.000
	NDVI	37.290	0.381	0.217
	Biome Area	43.461	0.404	0.115
	Isolation	44.838	0.423	0.034
	Temperature*Nitrogen	42.394	0.397	0.171
	Temperature*NDVI	29.845	0.329	0.415
	Temperature*Biome	31.512	0.337	0.387
	Temperature*Isolation	40.168	0.380	0.221
	Nitrogen*NDVI	42.675	0.398	0.164
	Nitrogen*Biome	40.642	0.387	0.211
	Nitrogen*Isolation	41.033	0.389	0.202
	NDVI*Biome	38.206	0.369	0.262
	NDVI*Isolation	40.539	0.382	0.014
	Temperature*Nitrogen*NDVI	35.336	0.318	0.465
	Temperature*Nitrogen*Biome	37.587	0.329	0.430
	**Temperature*NDVI*Biome**	**29.489**	**0.292**	**0.538**
	Temperature*NDVI*Isolation	37.256	0.327	0.001
Invertebrates	Intercept only	47.515	0.472	NA
	Temperature above ambient	40.084	0.413	0.234
	Nitrogen	46.898	0.477	0.000
	NDVI	46.396	0.455	0.073
	Biome Area	49.128	0.479	0.000
	Isolation	48.135	0.467	0.023
	Temperature*Nitrogen	40.748	0.410	0.247
	**Temperature*NDVI**	**28.154**	**0.329**	**0.516**
	Temperature*Biome	34.114	0.360	0.420
	Temperature*Isolation	37.416	0.378	0.359
	Nitrogen*NDVI	44.230	0.433	0.158
	Nitrogen*Biome	43.145	0.426	0.187
	Nitrogen*Isolation	38.888	0.398	0.291
	NDVI*Biome	51.518	0.468	0.017
	NDVI*Isolation	41.896	0.405	0.266
	Temperature*Nitrogen*NDVI	30.154	0.298	0.602
	Temperature*Nitrogen*Biome	30.039	0.297	0.604
	Temperature*NDVI*Biome	38.755	0.338	0.487
	Temperature*NDVI*Isolation	38.667	0.338	0.489

Supplementary Figure 4: Trends in beta diversity. *Turnover in the* ***(a)*** *diatom and* ***(b)*** *invertebrate communities with increasing temperature difference between streams within each region. Symbols represent a single stream, and coloured lines are negative exponential GLMs for each region.*

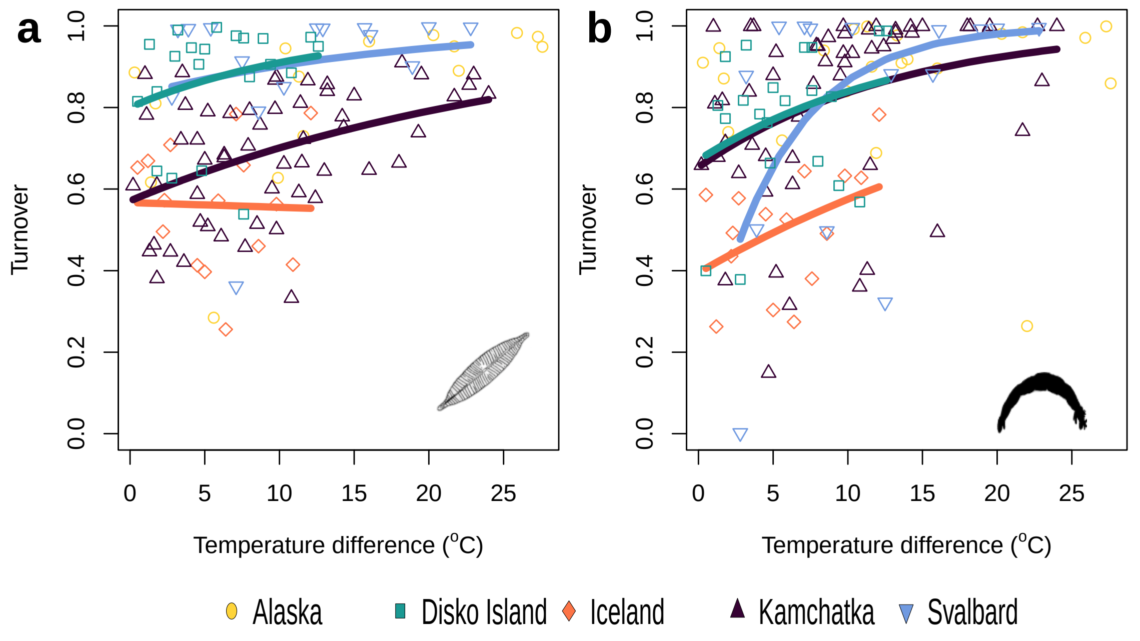

Supplementary Table 3: *Analysis of bootstrapped temperature-turnover relationships for each region. Models were specified as negative exponential equations.*

**Region**	**Group**	**df**	**Slope**	***P***	**R^2**
Alaska	Diatoms	1,13	0.057	0.055	0.293
	Invertebrates	1,19	0.000	0.991	0.000
Disko Island	Diatoms	1,19	0.079	0.270	0.069
	Invertebrates	1,19	0.072	0.212	0.090
Iceland	Diatoms	1,13	-0.003	0.930	0.001
	Invertebrates	1,13	0.035	0.030	0.196
Kamchatka	Diatoms	1,53	0.036	0.001	0.194
	Invertebrates	1,53	0.075	0.010	0.135
Svalbard	Diatoms	1,13	0.059	0.439	0.060
	Invertebrates	1,13	0.190	0.130	0.236

Supplementary Table 4: *Comparison of models of temperature-turnover slopes as a function of taxonomic group and biogeographic variables.*

**Model**	**Variable**	**df**	**F-value**	***P***	**AICc**
Slope ~ Biome*Group	Biome	1	0.776	0.412	-9.34
	Group	1	0.655	0.449	
	Biome*Group	1	0.929	0.372	
	Residuals	6			
Slope ~ NDVI*Group	NDVI	1	5.703	0.054	-14.40
	Group	1	1.086	0.338	
	NDVI*Group	1	1.078	0.339	
	Residuals	6			
Slope ~ Isolation*Group	Isolation	1	0.177	0.689	-9.28
	Group	1	0.651	0.451	
	Isolation*Group	1	1.482	0.270	
	Residuals	6			

***Supplementary* Table 5:** Results of our Detrended Correspondence Analysis (DCA) showing which environment variables correspond with each axis.

Group	Variable	DCA1	DCA2	r^2	P
Diatoms	Temperature	-0.053	-0.999	0.678	0.001
	pH	0.094	0.996	0.341	0.003
	Conductivity	0.006	-1.000	0.133	0.099
	NDVI	0.587	-0.810	0.346	0.001
	Isolation	0.965	0.262	0.473	0.001
	Area	-0.458	-0.889	0.296	0.002
	Nitrogen	-0.788	-0.616	0.267	0.012
Invertebrates	Temperature	-0.936	0.353	0.288	0.01
	pH	0.881	-0.473	0.124	0.153
	Conductivity	-0.304	0.953	0.121	0.116
	NDVI	-0.128	-0.992	0.075	0.295
	Isolation	0.641	0.767	0.182	0.047
	Area	-0.908	-0.419	0.254	0.023
	Nitrogen	-0.860	0.511	0.244	0.013

SUPPLEMENTARY REFERENCES

1. Krammer, K. & Lange-Bertalot, H. Bacillariophyceae 1. Teil: Naviculaceae. In Süβwasserflora von Mitteleuropa; Ettl, H., Gerloff, J., Heynig, H., Mollenhauer, D., Eds.; Gustav Fischer Verlag: Stuttgart, Germany, 1986.

2. Krammer, K. & Lange-Bertalot, H. Bacillariophyceae 2. Teil: Bacillariaceae, Epithemiaceae, Surirellaceae. In Süβwasserflora von Mitteleuropa; Ettl, H., Gerloff, J., Heynig, H., Mollenhauer, D., Eds.; Gustav Fischer Verlag: Stuttgart, Germany, 1988.

3. Krammer, K. & Lange-Bertalot, H. Bacillariophyceae 3. Teil: Centrales, Fragilariaceae, Eunotiaceae. In Süβwasserflora von Mitteleuropa; Ettl, H., Gerloff, J., Heynig, H., Mollenhauer, D., Eds.; Gustav Fischer Verlag: Stuttgart, Germany, 1991.

4. Krammer, K. & Lange-Bertalot, H. Bacillariophyceae 4. Teil: Achnanthaceae, Kritische Ergänzungen zu Navicula (Lineolatae) und Gomphonema. In Süβwasserflora von Mitteleuropa; Ettl, H., Gerloff, J., Heynig, H., Mollenhauer, D., Eds.; Gustav Fischer Verlag: Stuttgart, Germany, 1991.

5. Krammer, K. Diatoms of Europe, Volume 1: The genus Pinnularia. In Diatoms of the European Inland Waters and Comparable Habitats; Lange-Bertalot, H., Ed.; Gantner Verlag: Ruggell, Liechtenstein, 2000.

6. Krammer, K. Diatoms of Europe, Volume 4: Cymbopleura, Delicata, Navicymbula, Gomphocymbellopsis, Afrocymbella. In Diatoms of the European Inland Waters and Comparable Habitats; Lange-Bertalot, H., Ed.; Gantner Verlag: Ruggell, Liechtenstein, 2003.

7. Krammer, K. Diatoms of Europe, Volume 3: The genus Cymbella. In Diatoms of the European Inland Waters and Comparable Habitats; Lange-Bertalot, H., Ed.; Gantner Verlag: Ruggell, Liechtenstein, 2002.

8. Lange-Bertalot, H. & Metzeltin, D. Oligotrophie-Indikatoren: 800 Taxa repräsentativ für drei diverse Seen-Typen, kalkreich-oligodystroph-schwach gepuffertes Weichwasser. In Iconographia Diatomologica; Lange-Bertalot, H., Ed.; Koeltz Scientific Books, Schmitten: Oberreifenberg, Germany, 1996.

9. Lange-Bertalot, H. Diatoms of Europe, Volume 2: Navicula sensu stricto, 10 genera separated from Navicula sensu lato, Frustulia. In Diatoms of the European Inland Waters and Comparable Habitats; Lange-Bertalot, H., Ed.; Ganthner Verlag: Ruggell, Liechtenstein, 2011.

10. Lange-Bertalot, H., Bak, M., Witkowski, A. & Tagliaventi, N. Diatoms of Europe, Volume 6: Eunotia and some related genera. In Diatoms of the European Inland Water and Comparable Habitats; Krammer, K., Ed.; Ganthner
11. Verlag: Ruggell, Liechtenstein, 2011.

12. Lange-Bertalot, H., Hofmann, G., Werum, M. & Cantonati, M. Freshwater Benthic Diatoms of Central Europe: Over 800 Common Species Used in Ecological Assessment. In Koeltz Botanical Books; Cantonati, M., Kelly, M.G. & Lange–Bertalot, H., Eds.; Schmitten: Oberreifenberg, Germany, 2017.

13. Brinkhurst, R.O. (1963) Guide for the Identification of British Aquatic Oligochaeta. Freshwater Biological Association Scientific Publication, No. 22. 55 pp.

14. Brooks, S.J., Langdon, P.G. and Heiri, O. (2007) The identification and use of Palaearctic Chironomidae larvae in palaeoecology. QRA Technical Guide No. 10. London, Quaternary Research Association. 276 pp.

15. Colloff, M.J. (1993) A taxonomic revision of the oribatid mite genus Camisia (Acari: Oribatida). Journal of Natural History. 27(6): 1325-1408. https://doi.org/10.1080/00222939300770761.

16. Digital Key To Aquatic Insects of North Dakota (online). Available from: https://www.waterbugkey.vcsu.edu/index.htm (accessed: 2017). Valley City State University, Macro-Invertebrate Lab.

17. Friday, L.E. (1988) A Key to the adults of British Water Beetles. Field Studies. 7: 1-151.

18. Gelhaus, J. K. (2002) Manual for the identification of aquatic crane fly larvae for Southeastern United States. Durham, North Carolina (USA): Carolina Area Benthological Workshop. 205 pp.

19. Haney, J.F. et al. (2013) An-Image-based Key to the Zooplankton of North America. [Online], Version 5.0, released 2013. University of New Hampshire, Center for Freshwater Biology. Available from: http://cfb.unh.edu/cfbkey/html/index.html (accessed: 2017).

20. Hudson, P.L. and Lesko, L.T. (2003) Free-living and Parasitic Copepods of the Laurentian Great Lakes: Keys and Details on Individual Species. Ann Arbor, MI: Great Lakes Science Center Home Page. Available from: http://www.glsc.usgs.gov/greatlakescopepods/ (accessed: 2017).

21. Janssens, F. (2017) Checklist of the collembola. Available from: http://www.collembola.org/doc/about.htm (accessed: 2017).

22. Johanssen, O.A. (1969) Aquatic Diptera. Eggs, Larvae, and Pupae of aquatic flies. Los Angeles (USA): Entomological Reprint Specialists.

23. Kettle, D.S. and Lawson, J.W.H. (1952) The early stages of British biting midges Culicoides latreille (Diptera: Ceratopogonidae) and allied genera. Bulletin of Entomological Research. 43(3): 421-467.

24. Langton, P.H. (1991) A key to pupal exuviae of West Palaearctic Chironomidae. University of Amsterdam (The Netherlands): The Expert Center for Taxonomic Identification (ETI). 386 pp.

25. McAlpine, J.F., Peterson, B.V., Shewell, G.E., Teskey, H.J., Vockeroth, J.R. and Wood, D.M. (eds.) (1981) Manual of Nearctic Diptera Vol. 1-2-3. Research Branch, Agriculture Canada no. 27. Minister of Supply and Services Canada.

26. Morse, J.C. (ed.) (2021) Trichoptera World Checklist. Available from: http://entweb.clemson.edu/database/trichopt/index.htm (accessed: 2017).

27. Nilsson, A. (ed.) (1996) Aquatic Insects of North Europe. A taxonomic handbook. Vol. 1: Ephemeroptera. Plecoptera, Heteroptera, Magaloptera, Neuroptera, Coleoptera, Trichoptera and Lepidoptera. Stenstrup (Denmark): Apollo Books Aps. 274 pp.

28. Nilsson, A. (ed.) (1997) Aquatic insects of Northern Europe. A taxonomic handbook, Vol. 2: Odonata. Diptera. Stenstrup (Denmark): Apollo Books Aps. 440 pp.

29. Ronderos, M.M, Spinelli, G.R. and Diaz, F. (2004) Description of Larva and redescription of Pupa and Adult of Palpomyia guarani (Diptera: Ceratopogonidae). Revista de la Sociedad Entomológica Argentina. 63(1-2): 45-54.

30. Ronderos, M.M, Spinelli, G.R. and Diaz, F. (2007) Redescription of the last instar larva and pupa of Bezzia roldani (Diptera: Ceratopogonidae). Revista de la Sociedad Entomológica Argentina. 66(1-2): 21-28.

31. Seniczaka, S., Norton, R.A. and Seniczak, A. (2009) Morphology of Hydrozetes confervae (Schrank, 1781) and H. parisiensis Grandjean, 1948 (Acari: Oribatida: Hydrozetidae), and keys to European species of Hydrozetes Berlese, 1902. Zoologischer Anzeiger. 248(2): 71–83. https://doi.org/10.1016/j.jcz.2009.01.001.

32. Smith, K.G.V. (1989) Handbooks for the Identification of British Insects. Vol. 10, Part 14: An Introduction to the Immature Stages of British Flies. Diptera Larvae, with notes on eggs, puparia and pupae. London, Royal Entomological Society of London. 166 pp.

33. Tofilski, A. (2008-2013) DrawWing for insects identification [online]. Available from: http://drawwing.org/node/1 (accessed: 2017).

34. Thorp, J. and Rogers, D.C. (eds.) (2014) Ecology and General Biology: Thorp and Covich's Freshwater Invertebrates. Fourth Edition. Cambridge, Massachusetts, Academic Press. 1148 pp.

35. Thyssen P.J. (2009) Keys for Identification of Immature Insects. In: Amendt J., Goff M., Campobasso C., Grassberger M. (eds) Current Concepts in Forensic

Entomology. Dordrecht (The Netherlands): Springer. https://doi.org/10.1007/978-1-4020-9684-6_2.

36. Walter, T.C. and Boxshall, G. (2021) World of Copepods Database [online]. Available from: http://www.marinespecies.org/copepoda (accessed: 2017).

37. Wiederholm, T. (ed.) (1983) Chironomidae of the Holarctic Region: Keys and Diagnoses, Part 1: Larvae. Entomologica Scandinavica, Supplement 19, vol. 66. 457 pp.

38. Brinkhurst, R.O. (1986) Guide to the Freshwater Aquatic Microdrile Oligochaetes of North America. Canadian Special Publication of Fisheries and Aquatic Sciences. 84. 259 p.

39. Burch, J.B. (1982) Freshwater Snails (Mollusca: Gastropoda) of North America. Cincinnati, Ohio, Environmental Protection Agency.

40. Chapman, J.W. (2007) Amphipoda. In: Carlton, J.T. (ed.) The Light and Smith Manual. Intertidal Invertebrates from Central California to Oregon. Fourth Edition. University of California Press. 1019 pp.

41. Danks, H.V. and Downes, J.A. (eds.) (1997) Insects of the Yukon. Ottawa, Biological Survey of Canada (Terrestrial Arthropods). 1034 pp.

42. Edmunds, G.F. Jr., Jensen, S.L. and Berner, L. (1976) The Mayflies of North and Central America. Minnesota (USA), University of Minnesota Press. 344 pp.

43. Hagele, T., Kaufman, B., Whitaker, J.O. Jr. and Klompen, H. (2005) The genus Euryparasitus in North America (Mesostigmata: Euryparasitidae). Zootaxa. 1036: 1-20.

44. Hudson, P.L. and Lesko, L.T. (2003) Free-living and Parasitic Copepods of the Laurentian Great Lakes: Keys and Details on Individual Species. Ann Arbor, MI: Great Lakes Science Center Home Page. Available from: http://www.glsc.usgs.gov/greatlakescopepods/ (accessed: 2017).

45. Hudson, J., Hocker, K. and Armstrong, R.H. (2012) Aquatic Insects in Alaska. Juneau (Alaska), Nature Alaska Images. 140 pp.

46. Kenk, R. (1953) The Fresh-water Triclads (Turbellaria) of Alaska. Proceedings of the United States National Museum. 103 (3322): 163-186.

47. Merritt, R.W., Cummins, K.W. and Berg, M.B. (1995) An Introduction to Aquatic Insects of North America. Third edition. Dubuque, Iowa (USA), Kendall Hunt Pub. Co. 861 pp.

48. Proctor, H. (2006) Key to Aquatic Mites Known from Alberta. Available from: http://www.biology.ualberta.ca/faculty/heather_proctor/uploads/pdfs/AquaticMitesKnown_Alberta%20Aug_2006.pdf (accessed: 2017).

49. Randolph, R.P. (2002) Atlas and biogeographic review of the North American mayflies (Ephemeroptera). PhD dissertation, Purdue University, Indiana (USA).

50. Randolph, R.P. (2005) The mayflies (Ephemeroptera) of Alaska, including a new species of Heptageniidae. Proceedings of the Entomological Society of Washington. 107(1): 190-199.

51. Schmid, F. (1998) The insects and Arachnids of Canada, Part 7: Genera of the Trichoptera of Canada and Adjoining or Adjacent American States. Ottawa (Canada), Canadian Science Publishing (NRC Research Press). 320 pp.

52. Sikes, D., Coon, C. and Bowser, M. (2009) Export of a checklist of the terrestrial arthropods of Alaska. Available from: https://www.naturebob.com/sites/default/files/insect%20checklist%20for%20Alaska.pdf (accessed: 2017).

53. Stewart, K.W. and Oswood, M. (2006) The Stoneflies (Plecoptera) of Alaska and Western Canada. Columbus, Ohio (USA), The Caddis Press. 325 pp.

54. Stewart, K.W. and Stark, B.P. (2002) Nymphs of North American Stonefly Genera (Plecoptera). Second edition. Columbus, Ohio (USA), The Caddis Press. Xii + 510 pp.

55. Stone, A. (1952) The Simuliidae of Alaska. Proceedings of the Entomological Society of Washington. 54(2): 69-96.

56. Thorp, J.H. and Covich, A. (eds.) (2010) Ecology and Classification of North American Freshwater Invertebrates. Third edition. Cambridge (USA), Academic Press Elsevier. 1021 pp.

57. Thorp, J.H. and Rogers, C. (eds.) (2010) Field Guide to Freshwater Invertebrates of North America. Cambridge (USA), Academic Press Elsevier. 304 pp.

58. Walter, D.E. (2008) Key to Fully Aquatic Oribatid Mites in Alberta. Available from: http://www.biology.ualberta.ca/faculty/heather_proctor/uploads/pdfs/Key_to_Aquatic_Oribatida_from_Alberta.pdf (accessed: 2017).

59. Wiggins, G. (1996) Larvae of the North American Caddisfly Genera (Trichoptera). Second edition. Toronto (Canada), University of Toronto Press Incorporated. 472 pp.

60. Böcher, J., Kristensen, N.P., Pape, T. and Vilhelmsen, L. (eds.) (2015) The Greenland Entomofauna. An identification manual of Insects, Spiders and Their Allies. Fauna Entomologica Scandinavica. Vol. 44. Leiden (The Netherlands), Brill Publishers. 881 pp.

61. Makarova, O.L. (2015) The fauna of free-living mites (Acari) of Greenland. Entomological Review. 95(1): 108–125.

62. Hrafnsdottir, T. (2005) Diptera 2: Chironomidae. In: Gíslason, G.M., Jónasson, P. and Ottóson, J.G. (eds.) The Zoology of Iceland. Vol. 3, Part 48b. Steenstrupia. 1-167.

63. Hrafnsdottir, T. (2005) Diptera 2: Chironomidae. In: Gíslason, G.M., Jónasson, P. and Ottóson, J.G. (eds.) The Zoology of Iceland. Vol. 3, Part 48b. Steenstrupia. 1-167.

64. Rossaro, B. and Lencioni, V. (2015) A key to larvae of Diamesa Meigen, 1835 (Diptera,
65. Chironomidae), well known as adult males and pupae from Alps (Europe). Journal of
66. Entomological and Acarological Research. 47 (5516): 123-138. https://doi.org/10.4081/jear.2015.5516

67. Schulze, E. (1994) A Key to the Larval Chironomidae and their Instars from Austrian Danube Region Streams and Rivers with Particular Reference to a Numerical Taxonomic Approach. Part I. In: Wasser und Abwasser, Supplementband 3/93. Hrsg.: Bundesamt für Wassergüte, Wien-Kaisermühlen. Schriftenleitung: Werner Kohl. Selbstverlag, 1993, 514 S., öS 562.

68. Behan-Pelletier, V.M. and Norton, R.A. (1983) Epidamaeus (Acari: Damaeidae) of arctic western north America and extreme northeast, U.S.S.R. The Canadian Entomologist. 115(10): 1253-1289.

69. Conservation of biodiversity of Kamchatka and coastal waters (2013). Abstracts of the XIV international scientific conference, dedicated to the 100th anniversary of V.Ya. Levanidov’s birthday. Petropavlovsk-Kamchatsky: Kamchatpress. 426 p.

70. Komai, T. (2000) Results of recent research on northeast Asian biota. Natural History Research Special Issue. 7. Natural History Museum and Institute, Chiba (Japan).

71. Kurenkov, I.I. (1967) List of aquatic invertebrates of internal waters in Kamchatka. Bulletin of the Pacific Scientific Research Institute of Fisheries and Oceanography. 57: 202-224. [In Russian].

72. Lepneva, S.G. (1970) Fauna of the U.S.S.R.: Trichoptera. Larvae and Pupae of Annulipalpia. Program for Scientific Translations, Jerusalem (Israel). 652 pp.

73. Makarchenko, M.A., Makarchenko, E.A. and Vvedenskaja, T.L. (1997) A preliminary chironomid list (Diptera, Chironomidae) of the Kamchatka peninsula and bordering territories. Far Eastern Entomologist. 40, 1-7.

74. Tiunova, T.M. (2009) Biodiversity and distribution of mayflies (Ephemeroptera) in the Russian Far East. Aquatic Insects. 31, Sup. 1: 671-691. http://dx.doi.org/10.1080/01650420902800581

75. Tsalolikhin, S.J. (ed.) (1997) Key to freshwater invertebrates of Russia and adjacent lands. Vol. 3. Arachnids, Lower insects. St. Petersburg (Russia), Institute of Zoology of the Russian Academy of Sciences [In Russian].

76. Tsalolikhin, S.J. (ed.) (2000) Key to freshwater invertebrates of Russia and adjacent lands. Vol. 4. Higher insects: Diptera. St. Petersburg (Russia), Institute of Zoology of the Russian Academy of Sciences [In Russian].

77. Tsalolikhin, S.J. (ed.) (2001) Key to freshwater invertebrates of Russia and adjacent lands. Vol. 5. Higher insects: Trichoptera, Lepidoptera, Coleoptera, Neuroptera, Megaloptera, Hymenoptera. St. Petersburg (Russia), Institute of Zoology of the Russian Academy of Sciences [In Russian].

78. Zaitsev, F.A. (1972) Fauna of the U.S.S.R.: Coleoptera. Volume 4. Families Amphizoidae, Hygrobiidae, Haliplidae, Dytiscidae, Gyrinidae. Program for Scientific Translations, Jerusalem (Israel). 416 pp.

79. Zasypkina, I.A. and Ryabukhin, A.S. (2001) Amphibiotic insects of the northeast of Asia. Sofia (Bulgaria), Pensoft & Backhuys publishers BV. 183 pp.

80. Coulson, S.J. (2009-2021) SPIDER (Svalbard Pictographic Invertebrate Database Educational Resource). Available from: http://svalbardinsects.net (accessed: 2017).

81. Coulson, S.J. (2013) The terrestrial and freshwater invertebrate fauna of Svalbard: a checklist. [online]. Available from: https://www.yumpu.com/en/document/read/16680115/checklist-of-the-terrestrial-and-freshwater-invertebrate-fauna-unis

82. Kotwicki, L. (2002) Benthic Harpacticoida (Crustacea, Copepoda) from the Svalbard archipelago. Polish Polar Research. 23(1): 185–191.
