# Supplementary material for: Regional impacts of warming on biodiversity and biomass in high latitude stream ecosystems across the Northern Hemisphere
Source: Commun Biol. 2024 Mar 13;7:316. doi: 10.1038/s42003-024-05936-w (PMC10937648; doi:10.1038/s42003-024-05936-w)
Supplement: Supplementary file 2 — Reporting Summary [file 42003_2024_5936_MOESM2_ESM.pdf]

nature portfolio

Corresponding author(s): Michelle Jackson

Last updated by author(s): 30.11.23

Reporting Summary

Nature Portfolio wishes to improve the reproducibility of the work that we publish. This form provides structure for consistency and transparency in reporting. For further information on Nature Portfolio policies, see our Editorial Policies and the Editorial Policy Checklist.

Statistics

For all statistical analyses, confirm that the following items are present in the figure legend, table legend, main text, or Methods section.

n/a	Confirmed	
☐	☒	The exact sample size (*n*) for each experimental group/condition, given as a discrete number and unit of measurement
☐	☒	A statement on whether measurements were taken from distinct samples or whether the same sample was measured repeatedly
☐	☒	The statistical test(s) used AND whether they are one- or two-sided<br>*Only common tests should be described solely by name; describe more complex techniques in the Methods section.*
☐	☒	A description of all covariates tested
☐	☒	A description of any assumptions or corrections, such as tests of normality and adjustment for multiple comparisons
☐	☒	A full description of the statistical parameters including central tendency (e.g. means) or other basic estimates (e.g. regression coefficient) AND variation (e.g. standard deviation) or associated estimates of uncertainty (e.g. confidence intervals)
☐	☒	For null hypothesis testing, the test statistic (e.g. *F*, *t*, *r*) with confidence intervals, effect sizes, degrees of freedom and *P* value noted<br>*Give P values as exact values whenever suitable.*
☒	☐	For Bayesian analysis, information on the choice of priors and Markov chain Monte Carlo settings
☒	☐	For hierarchical and complex designs, identification of the appropriate level for tests and full reporting of outcomes
☒	☐	Estimates of effect sizes (e.g. Cohen's *d*, Pearson's *r*), indicating how they were calculated

Our web collection on statistics for biologists contains articles on many of the points above.

Software and code

Policy information about availability of computer code

Data collection	No software used in data collection
Data analysis	All analyses were conducted in R (R Core Team 2019) with the following packages: MODISTools, Vegan, betapart.

For manuscripts utilizing custom algorithms or software that are central to the research but not yet described in published literature, software must be made available to editors and reviewers. We strongly encourage code deposition in a community repository (e.g. GitHub). See the Nature Portfolio guidelines for submitting code & software for further information.

Data

Policy information about availability of data

All manuscripts must include a data availability statement. This statement should provide the following information, where applicable:

- Accession codes, unique identifiers, or web links for publicly available datasets
- A description of any restrictions on data availability
- For clinical datasets or third party data, please ensure that the statement adheres to our policy

All data ahave been uploaded to FigShare as part of the submission process

Research involving human participants, their data, or biological material

Policy information about studies with human participants or human data. See also policy information about sex, gender (identity/presentation), and sexual orientation and race, ethnicity and racism.

Reporting on sex and gender	*Use the terms sex (biological attribute) and gender (shaped by social and cultural circumstances) carefully in order to avoid confusing both terms. Indicate if findings apply to only one sex or gender; describe whether sex and gender were considered in study design; whether sex and/or gender was determined based on self-reporting or assigned and methods used. Provide in the source data disaggregated sex and gender data, where this information has been collected, and if consent has been obtained for sharing of individual-level data; provide overall numbers in this Reporting Summary. Please state if this information has not been collected. Report sex- and gender-based analyses where performed, justify reasons for lack of sex- and gender-based analysis.*
Reporting on race, ethnicity, or other socially relevant groupings	*Please specify the socially constructed or socially relevant categorization variable(s) used in your manuscript and explain why they were used. Please note that such variables should not be used as proxies for other socially constructed/relevant variables (for example, race or ethnicity should not be used as a proxy for socioeconomic status). Provide clear definitions of the relevant terms used, how they were provided (by the participants/respondents, the researchers, or third parties), and the method(s) used to classify people into the different categories (e.g. self-report, census or administrative data, social media data, etc.) Please provide details about how you controlled for confounding variables in your analyses.*
Population characteristics	*Describe the covariate-relevant population characteristics of the human research participants (e.g. age, genotypic information, past and current diagnosis and treatment categories). If you filled out the behavioural & social sciences study design questions and have nothing to add here, write "See above."*
Recruitment	*Describe how participants were recruited. Outline any potential self-selection bias or other biases that may be present and how these are likely to impact results.*
Ethics oversight	*Identify the organization(s) that approved the study protocol.*

Note that full information on the approval of the study protocol must also be provided in the manuscript.

Field-specific reporting

Please select the one below that is the best fit for your research. If you are not sure, read the appropriate sections before making your selection.

☐ Life sciences ☐ Behavioural & social sciences ☒ Ecological, evolutionary & environmental sciences

For a reference copy of the document with all sections, see nature.com/documents/nr-reporting-summary-flat.pdf

Ecological, evolutionary & environmental sciences study design

All studies must disclose on these points even when the disclosure is negative.

Study description	Our study was conducted in five high latitude regions (Figure 1a) with geothermal areas nested within them to provide natural thermal gradients: Iceland (mean summer stream temperature of sampled streams: 5.8–20.3 °C), Kamchatka (Russia; 7.0–31.0 °C), Alaska (USA; 5.5–33.1 °C), Disko Island (Greenland; 1.7–14.3 °C), and Svalbard (1.5–24.3 °C; Table S1). Within each region, all streams share the same river basin (see Supporting Information). We sampled 6-11 streams per region, with an "ambient" reference stream in each as the baseline water temperature that reflects the prevailing above ground temperature.
Research sample	Invertebrates and diatoms from streams - the major eukaryotes in these systems.
Sampling strategy	We sampled benthic invertebrates from each stream using a Surber sampler (0.0225 m2, 250um mesh). Three technical replicate samples were collected from random locations in each stream and preserved in 70%. Diatoms were sampled by randomly selecting three stones which were then brushed using a toothbrush detaching algae while rinsing with spray bottle using water28. Algae samples were preserved in Lugols solution.
Data collection	All invertebrates were identified and counted using a Nikon SMZ800 at magnification of X10-63. For diatoms, From each sample, 400 randomly encountered diatom valves were determined to the lower taxonomical level possible using a Leica DM2500 light microscope, at a magnification of 1000.
Timing and spatial scale	Five locations in the Arctic and Sub-Arctic provides a large spatial scale. Each site was sampled once during summer months.
Data exclusions	NA
Reproducibility	NA
Randomization	All samples were taken randomly.

Blinding	NA

Did the study involve field work? ☒ Yes ☐ No

Field work, collection and transport

Field conditions	Streams were sampled in summer months with no specific conditions required for sampling to take place.
Location	There are 37 sites in the 5 regions described above - all 37 lat and longs are provided in the Supporting Information.
Access & import/export	Local scientists were included at all levels of the study, from design to collection and write up. Permits were obtained where needed (Svalbard, Kamchatka, Greenland) and local land owner permission was also ensured.
Disturbance	We reduced disturbance by using the same paths to the sites to minimise trampling. No other disturbance was caused.

Reporting for specific materials, systems and methods

We require information from authors about some types of materials, experimental systems and methods used in many studies. Here, indicate whether each material, system or method listed is relevant to your study. If you are not sure if a list item applies to your research, read the appropriate section before selecting a response.

Materials & experimental systems

n/a	Involved in the study	
☒	☐	Antibodies
☒	☐	Eukaryotic cell lines
☒	☐	Palaeontology and archaeology
☒	☐	Animals and other organisms
☒	☐	Clinical data
☒	☐	Dual use research of concern
☒	☐	Plants

Methods

n/a	Involved in the study	
☒	☐	ChIP-seq
☒	☐	Flow cytometry
☒	☐	MRI-based neuroimaging
